# Case report: A novel 10.8-kb deletion identified in the β-globin gene through the long-read sequencing technology in a Chinese family with abnormal hemoglobin testing results

**DOI:** 10.3389/fmed.2023.1192279

**Published:** 2023-07-13

**Authors:** Mingkun Shao, Yaoyao Wan, Weipeng Cao, Juan Yang, Di Cui, Minhui Ma, Wanqin Hu

**Affiliations:** ^1^Department of OB and GYN, The Second Affiliated Hospital of Kunming Medical University, Yunnan, China; ^2^Department of Cardiovascular Medicine, The Second People's Hospital of Honghe Autonomous Prefecture, Yunnan, China; ^3^Jinyu Medical Laboratory Co., Ltd., Yunnan, China; ^4^Berry Genomics Corporation, Beijing, China

**Keywords:** thalassemia, long-read sequencing (LRS), β-globin gene (*HBB*), large fragment deletion, multiplex ligation-dependent probe amplification (MLPA), gap-polymerase chain reaction

## Abstract

**Background:**

Thalassemia is a common inherited hemoglobin disorder caused by a deficiency of one or more globin subunits. Substitution variants and deletions in the *HBB* gene are the major causes of β-thalassemia, of which large fragment deletions are rare and difficult to be detected by conventional polymerase chain reaction (PCR)-based methods.

**Case report:**

In this study, we reported a 26-year-old Han Chinese man, whose routine blood parameters were found to be abnormal. Hemoglobin testing was performed on the proband and his family members, of whom only the proband's mother had normal parameters. The comprehensive analysis of thalassemia alleles (CATSA, a long-read sequencing-based approach) was performed to identify the causative variants. We finally found a novel 10.8-kb deletion including the β-globin (*HBB*) gene (Chr11:5216601-5227407, GRch38/hg38) of the proband and his father and brother, which were consistent with their hemoglobin testing results. The copy number and exact breakpoints of the deletion were confirmed by multiplex ligation-dependent probe amplification (MLPA) and gap-polymerase chain reaction (Gap-PCR) as well as Sanger sequencing, respectively.

**Conclusion:**

With this novel large deletion found in the *HBB* gene in China, we expand the genotype spectrum of β-thalassemia and show the advantages of long-read sequencing (LRS) for comprehensive and precise detection of thalassemia variants.

## Introduction

β-thalassemia is a hereditary hemolytic anemia caused by a deficiency of hemoglobin (Hb) synthesis due to the reduced or complete absence of β-globin subunit synthesis (1, 2). Genetic variants causing β-thalassemia are substitution variants or small fragment insertions/deletions in the *HBB* gene, and a few are large fragment deletions (2–4). β-thalassemia is distinctly geographic as well as population-specific. To date, more than 300 β-thalassemia variants have been identified around the world. However, more than 90% of the cases are caused by approximately 40 of these variants (2). In the Chinese population, 174 *HBB* variants (NM_000518.4) have been found (http://www.genomed.zju.edu.cn/LOVD3/genes), among which eight variants (c.-78A>G, c.-79A>G, c.52A>T, c.79G>A, c.92+1G>T, c.126_129delCTTT, c.216_217insA, and c.316-197C>T) account for approximately more than 95% of β-thalassemia cases in China (5). Therefore, the current clinical strategy is to first detect common substitution variants by reverse dot blot hybridization (RDB) or the PCR melting curve analysis (PMCA) method (6) and detect Chinese ^G^γ(^A^γ*δ*β)^0^-Thal deletion and Vietnamese HPFH deletion by gap-polymerase chain reaction (Gap-PCR) (3). If the diagnosis could not be confirmed, Sanger sequencing, multiplex ligation-dependent probe amplification (MLPA), array comparative genomic hybridization (array-CGH), or next-generation sequencing (NGS) technology were used to detect rare or unknown variants (7). However, these traditional PCR-based assays can only detect known hotspot variants, with a risk of approximately 5% of cases being missed (8). NGS technology can increase the detection of some substitution variants, but more deletions, triplet, and quadruplet variants cannot be detected. In recent years, a novel technology called single-molecule real-time (SMRT) based on long-read sequencing (LRS) has been established and used for the genetic analysis of thalassemia cases (9, 10). It provides direct access to the complete α- and β- gene sequences and can accurately detect not only unknown and structural variants but also gene homologous, repetitive, and GC-rich region variants. The haplotypes of two alleles can be analyzed, and the cis/trans configuration of two or more variants can be determined easily (11). In this study, we identified a novel 10.8-kb deletion in the *HBB* gene through LRS, which is further confirmed by MLPA and Gap-PCR methods.

## Methods

### Case presentation

The proband was a 26-year-old Han Chinese man. During a routine physical examination, his routine blood parameters were found to be abnormal (MCV: 63.5 fL; MCH: 21.6 pg), suggesting the possibility of thalassemia. The doctor suggested that he should undergo hemoglobin (Hb) testing to further confirm the diagnosis. He came to our hospital to undergo standard blood assays (Mindray 6800plus, China) and hemoglobin electrophoresis (Capillarys3TERA, Sebia, France), the results of which were abnormal (Table 1). Mean erythrocyte volume (MCV, 66.8 fL) and mean erythrocyte hemoglobin (MCH, 20.8 pg) levels were low. Hemoglobin electrophoresis showed lower than normal Hb A (89.7%) and higher than normal Hb A2 (7.3%) and Hb F (3.0%). The proband was suspected of having β-thalassemia minor (α-thalassemia complex cannot be excluded). Genetic testing for thalassemia and familial Hb analysis is recommended for a final diagnosis.

**Table 1:** Hematologic characteristics of the proband and his family members.

	**Normal range**	**Proband**	**Father**	**Mother**	**Brother**
RBC (/L)	4.3–5.8 × 10^12^	6.75 × 10^12^ ↑	5.26 × 10^12^	4.06 × 10^9^	6.20 × 10^12^ ↑
Hb (g/L)	130–175	140	124 ↓	124 ↓	134
HCT (%)	40.4–50.0	45.1	40.9	40.3	45.7
MCV (fL)	82.0–100.0	66.8 ↓	77.6 ↓	99.2	73.6 ↓
MCH (pg)	27.0–34.0	20.8 ↓	23.6 ↓	30.5	21.6 ↓
MCHC (g/L)	316–354	311 ↓	304 ↓	308 ↓	293 ↓
RDW	≤ 15.0	17.6 ↑	15.9	14.4	17.3↑
Hb A	>94.5	89.7 ↓	90.9 ↓	97.3	90.4 ↓
Hb A2	2.5–3.2	7.3 ↑	7.0 ↑	2.7	7.2 ↑
Hb F	0.0–2.0	3.0 ↑	2.1 ↑	0.0	2.4 ↑

RBC, red blood cell count; Hb, hemoglobin; HCT, hematocrit; MCV, mean corpuscular volume; MCH, mean corpuscular hemoglobin; MCHC, mean corpuscular hemoglobin concentration; RDW, red cell distribution width; arrow mark ↑, parameters that are higher than normal; arrow mark ↓, parameters that are lower than normal.

### Genetic analysis

The peripheral blood of the proband and his parents as well as his little brother was collected and sent to Berry Genomics Corporation for comprehensive analysis of thalassemia alleles (CATSA, a long-read sequencing-based approach) to identify the pathogenic variants as described previously (10). Briefly, DNA was isolated and amplified by multiplex long-range PCR with primers covering different types of currently known variants in the *HBA1, HBA2*, and *HBB* genes. After purification, end repair, and ligation with double-barcode adapters to both ends, PCR products were used to build Single-Molecule Real-Time (SMRT) bell libraries using the Sequel Binding and Internal Ctrl Kit 3.0 (Pacific Biosciences, Menlo Park, CA) and then were sequenced on a PacBio Sequel II platform. Raw subreads were demultiplexed and barcode sequences clipped using the *lima* in the Pbbioconda package (Pacific Biosciences) and were analyzed using the CCS software application to generate circular consensus sequencing (CCS) reads, which were then aligned to the GRCh38 reference using BLASR (12). The single nucleotide variations (SNVs) and small insertions and deletions (indels) were called by FreeBayes software (Biomatters, Inc., San Diego, CA). The structural variants were identified based on reads containing specific primer pairs, read length, and alignment to the reference sequence in the HbVar, Ithanet, and LOVD databases, as described in the previous literature (9). We only give a pass when variant/wild-type reads were >20% and total CCS reads were >100 for SNVs and indels, and when variant/wild-type reads were > 5% and total CCS reads were >50 for structural variants. General guidelines and hemoglobin variant databases were used to classify the pathogenicity of the identified variants (13–15). The candidate causal variants thereby discovered were then confirmed via multiple ligation-dependent probe amplification (MLPA, MRC Holland, Amsterdam, Netherlands), gap-polymerase chain reaction (Gap-PCR), and Sanger sequencing, according to the manufacturer's protocol (16).

## Results

**Figure 1:**
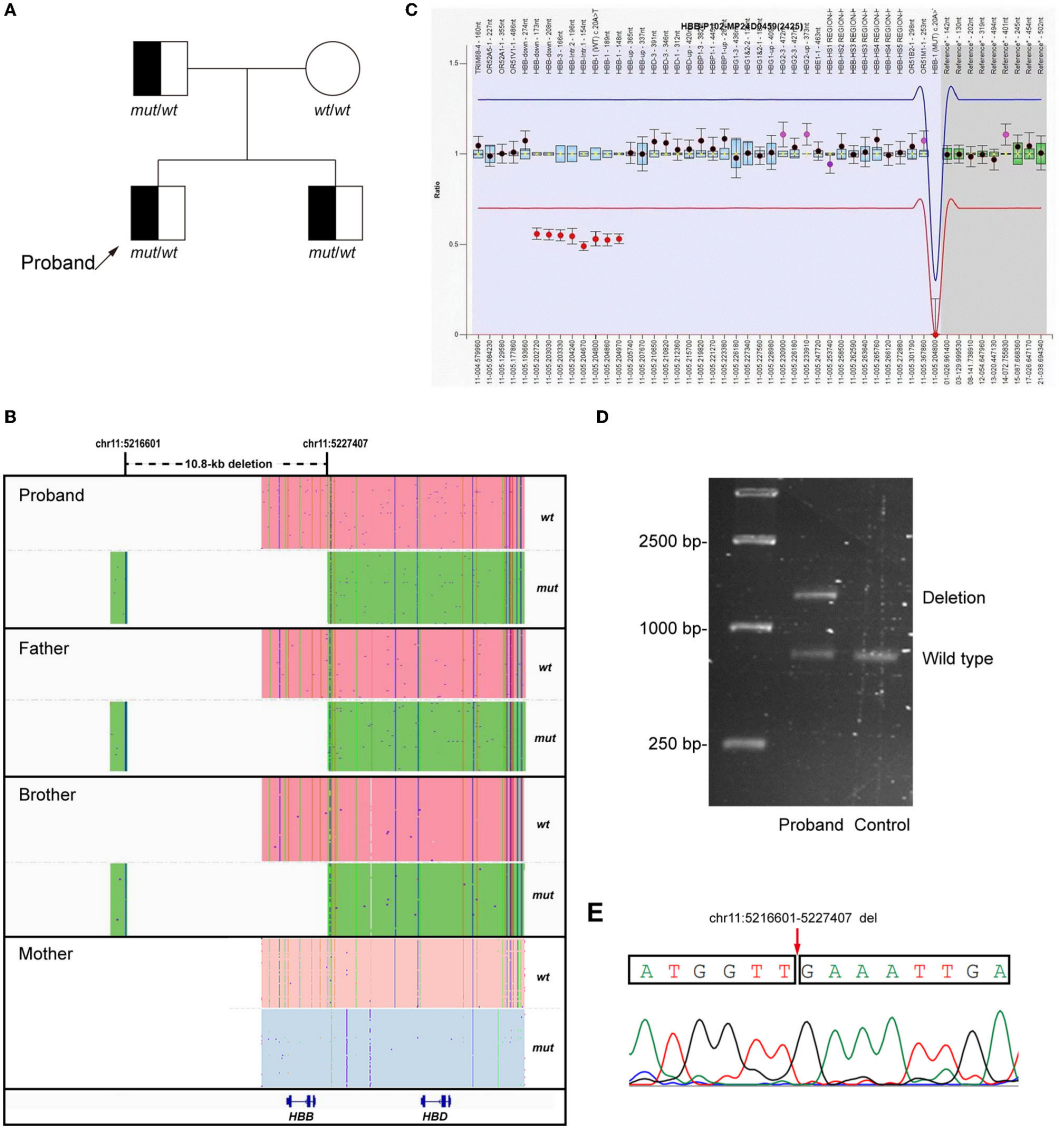
Genetic analysis of the proband. **(A)** Pedigree of the family. **(B)** LRS results showed a heterozygous 10.8-kb deletion including the *HBB* gene (Chr11:5216601-5227407, GRch38/hg38) in the proband and his father and brother. The pink and blue areas show the wild-type allele and the green area shows the allele with 10.8-kb deletion. **(C)** MLPA results suggested the deletion of all coding exons of *HBB*. **(D, E)** Gap-polymerase chain reaction (Gap-PCR), agarose gel electrophoresis, and Sanger sequencing confirmed the 10.8-kb deletion in the proband.

A novel heterozygous 10.8-kb deletion on chromosome 11 (chr11:5216601-5227407) was identified in the proband, which contained the whole *HBB* gene (Figure 1B). MLPA and Gap-PCR were then carried out to confirm the sequencing result. MLPA analysis showed half dosages for eight consecutive MLPA probes whose targets ranged from the promoter region to the downstream of *HBB*, suggesting deletion of all coding exons of *HBB* (Figure 1C). Gap-PCR and Sanger sequencing using specific primers (forward F1: 5'-GGCTCCTGTTTAGTATTGCTGCTTT-3'; forward F2: 5'-CCTCCCTGCTCCTGGGAGTAGATT-3'; reverse R: 5'-TGTCAAATAGGAGGTTAACTGGGGACA-3') confirmed the deletion (Figures 1D, E). The same variant was also identified in the proband's father and brother, indicating a variant transmission from the father to the two sons (Figures 1A, B). Results of hemoglobin testing, including standard blood and electrophoresis assays for the father and brother were abnormal and were similar to the proband's (Table 1). The mother of the proband showed no abnormalities in either genotype or biochemical findings. No other pathogenic variants were identified in the *HBA1* or *HBA2* genes of all family members. Therefore, the proband was finally diagnosed with β-thalassemia minor, and the 10.8-kb deletion of chromosome 11 was the cause of the disease.

## Discussion and conclusion

To date, more than 170 *HBB* variants have been identified in the Chinese population (http://www.genomed.zju.edu.cn/LOVD3/genes), most of which are substitution variants. In this study, we did not use traditional methods like suspension array system and PCR-reverse dot blot (PCR-RDB) to detect the causative variants, as the detection range is very limited: only six α-thalassemia deletions (^–SEA^, −α^3.7^, −α^4.2^, ^–THAI^, −α^21.9^, and −α^27.6^), three α-thalassemia SNVs, and 17 β-thalassemia SNVs were included (17). Rare deletions, triplets, quadruples, and other unreported variants could be missed. A long-read sequencing-based approach termed CATSA has been established for identifying both α- and β- thalassemia genetic carrier status, whose clinical utility has also been extended successfully to a diagnosis of thalassemia (9, 10, 18). A novel heterozygous 10.8-kb deletion on chromosome 11 was identified through CATSA and was confirmed by MLPA and Gap-PCR. The process of detecting substitution variants could be omitted, and we were able to use the Gap-PCR technique for verification (only known variants can be detected). We performed one experiment to detect all possible thalassemia variants, avoiding the cumbersome steps of combining multiple experimental techniques and reducing the risk of missed screening.

β-thalassemia is clinically heterogeneous and is divided into thalassemia major (transfusion-dependent), thalassemia intermedia (of intermediate severity), and thalassemia minor (asymptomatic and carrier state). Carriers usually have no clinical symptoms. Abnormal hematologic findings such as microcytic hypochromic red blood cell changes and mild anemia could be detected, which are often ignored because there is no clinical discomfort (19). If these “recessive” carriers want to have children, there is a 25% chance of giving birth to a child with thalassemia. In our case, the proband was found to have thalassemia during a routine physical examination. Genetic analysis was performed on the proband and his family members (his parents and brother). A deletion variant on chromosome 11 was identified, which was transmitted from the father to the proband and his brother. Each of the three men had only one *HBB* gene. The expression of the β-subunit was decreased, which disrupted the balance of the α- and β-globin subunits and caused their abnormal blood parameters. However, the clinical symptoms were so mild that the proband's father ignored it and did not consider the possibility of thalassemia. This suggests that if abnormalities are found in standard blood assay, clinicians should strongly recommend retesting and/or adding additional tests (hemoglobin electrophoresis and genetic screening, if necessary) to confirm the presence or carrier of thalassemia, especially when people are of marriageable and reproductive age.

In conclusion, we utilized LRS technology to identify a novel 10.8-kb deletion encompassing the whole *HBB* gene in a Chinese family, which led to abnormal hemoglobin testing results. Our study showed that LRS technology can rapidly, accurately, and comprehensively analyze the genotype of thalassemia in the proband, providing a powerful technical tool for diagnosis, carrier screening, and genotype-phenotype correlation studies of this disease.

## Data availability statement

The raw data supporting the conclusions of this article will be made available by the authors, without undue reservation.

## Ethics statement

The study was approved by the Ethics Committee of The Second Affiliated Hospital of Kunming Medical University (PJ-2022-170). A written informed consent was obtained from the participant for the publication of this case report (including all data and images).

## Author contributions

MS designed the study and wrote the original draft. YW cared for the proband and collected clinical data on the proband. WC and JY completed the hemoglobin testing. MM analyzed the results of LRS. DC modified the picture and revised the manuscript. WH finally revised the manuscript. All authors read and approved the submitted version.
